# Computational Fluid Dynamics Study of Swimmer's Hand Velocity, Orientation, and Shape: Contributions to Hydrodynamics

**DOI:** 10.1155/2013/140487

**Published:** 2013-04-09

**Authors:** Milda Bilinauskaite, Vishveshwar Rajendra Mantha, Abel Ilah Rouboa, Pranas Ziliukas, Antonio Jose Silva

**Affiliations:** ^1^Department of Mechanical Engineering, Kaunas University of Technology, LT-44029 Kaunas, Lithuania; ^2^Mechatronics Centre for Research, Studies and Information, Kaunas University of Technology, LT-44029 Kaunas, Lithuania; ^3^Department of Mechanical Engineering, University of Tras-os-Montes and Alto Douro, 5001-801 Vila Real, Portugal; ^4^Centre of Research in Sports, Health and Human Development, CIDESD, 5001-801 Vila Real, Portugal; ^5^Department of Sport Sciences, Exercise and Health, University of Tras-os-Montes and Alto Douro, 5001-801 Vila Real, Portugal; ^6^Department of Mechanical Engineering and Applied Mechanics, University of Pennsylvania, Philadelphia, PA 19104, USA

## Abstract

The aim of this paper is to determine the hydrodynamic characteristics of swimmer's scanned hand models for various combinations of both the angle of attack and the sweepback angle and shape and velocity of swimmer's hand, simulating separate underwater arm stroke phases of freestyle (front crawl) swimming. Four realistic 3D models of swimmer's hand corresponding to different combinations of separated/closed fingers positions were used to simulate different underwater front crawl phases. The fluid flow was simulated using FLUENT (ANSYS, PA, USA). Drag force and drag coefficient were calculated using (computational fluid dynamics) CFD in steady state. Results showed that the drag force and coefficient varied at the different flow velocities on all shapes of the hand and variation was observed for different hand positions corresponding to different stroke phases. The models of the hand with thumb adducted and abducted generated the highest drag forces and drag coefficients. The current study suggests that the realistic variation of both the orientation angles influenced higher values of drag, lift, and resultant coefficients and forces. To augment resultant force, which affects swimmer's propulsion, the swimmer should concentrate in effectively optimising achievable hand areas during crucial propulsive phases.

## 1. Introduction

The swimming propulsion is the result of the interaction of applied forces with water and is predominantly attributed to muscular force applied by hands and forearms. It was emphasised that the major part of about 85% to 90% of propulsion generation in water is created by the application of force by an arm [1, 2]. It was suggested that, the shoulder has the least propulsive potential, the forearm and hand approximately equal according to linear and angular velocities which are least by the shoulder and greater by the forearm and hand [3]. Miller [3] used the mathematical model of the front crawl arm pull with bending elbow and showed that the ratio of hydrodynamic forces of hand was about 2.5 bigger when compared with forearm. Toussaint and Truijens [4] studied the visualization of flow tufts around arm and hand. They showed that a strong pressure gradient along the arm occurred that induced axial flow directed from elbow to the hand. Apart from that, the biggest influence of pressure (relative to atmospheric pressure) was identified on the palm of hand when swimming at sprint speed. The authors noted that the pressure was not corrected for differences in hydrostatic pressure due to differences in the depth of the sensors.

**Figure 1:**
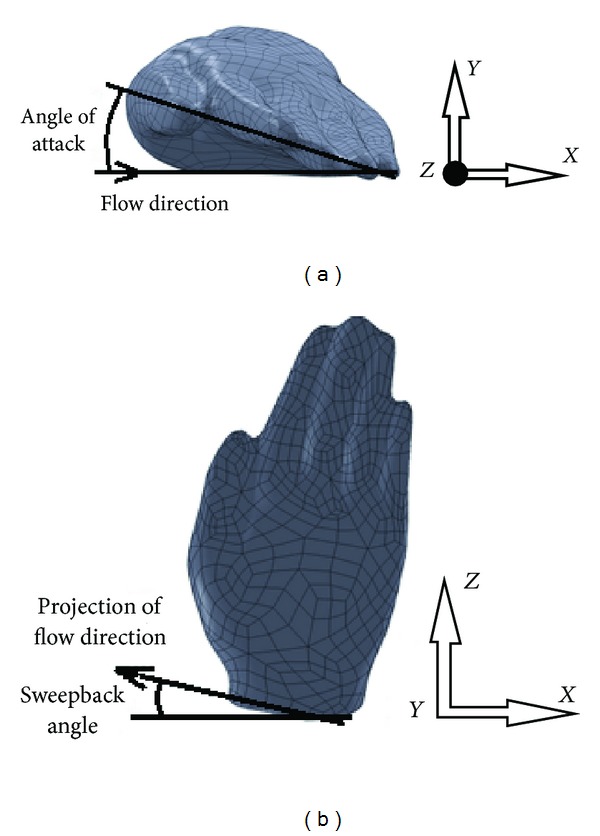
Orientations of swimmer's hand model: (a) angle of attack; (b) sweepback angle.

In reality, swimmers can change the depth, orientation, shape, and velocity of hand throughout underwater front crawl cycle. All these parameters have direct influence on propulsion force. The prevailing theory of propulsion generation relates to Newton's second and third laws of motion where propulsion is the vector sum of drag (*F*_*D*_: force opposite to the direction of fluid flow) and lift (*F*_*L*_: forces that are perpendicular to the fluid flow) forces [4, 5]. These components depend on the density of the fluid, the velocity of the limb relative to the fluid, the projected surface area of the limb, and the coefficient of drag (*C*_*D*_: a dimensionless constant used to show the resistance of the object in a fluid environment) and lift (*C*_*L*_) which vary according to the shape of the limb and its orientation (i.e., the angle of attack (or pitch angle) and the sweepback angle) (Figure 1). To uncover an influence of various parameters influencing propulsive force or its components, experimental studies [6–8] as well as numerical studies were carried out in the past [5, 9–12].

One of the approachs to calculate and assess forces acting on swimmer or on separated body segments is by an application of computational fluid dynamics (CFD) method. This methodology allows the analysis of the water flow with a reduced amount of complexity and is economical alternative to an experimental method. The validity studies of CFD results are usually carried out with comparative experimental studies. Gardano and Dabnichki [7] used replica of the entire human arm to assess drag and lift coefficients, measured in a low speed wind tunnel based experiment. The computational hand model was created from human body similar to experimental arm model. Authors showed that lift and drag coefficients obtained in wind tunnel experiments were in good correlation with CFD obtained from CFD simulations carried out using FLUENT (ANSYS, PA, USA). In similar lines, Bixler and Riewald [5] simulated the steady flow around a swimmer's hand and arm at various angles of attack in steady state by using CFD method. They improved validity of calculation and demonstrate that the force coefficients computed for the hand and arm compared well with steady-state coefficients determined experimentally by the previous studies.

In consideration of significant improvement in the methodology and validity of the CFD studies, this method is applied more often in swimming investigations. The calculations through CFD method were carried out to analyse the propulsive forces produced by the propelling segments and the drag force resisting forward motion. Rouboa et al. [11] calculated drag and lift coefficients and drag force for steady flow around a 2D swimmer's hand/forearm model placed at different angles of attack with application CFD technique. These results were compared with previous CFD studies and experimental results, which illustrate similar values under the steady-state flow conditions. It was explained that the increase in flow velocity did not have much influence on the variation of drag coefficient when the model of hand/forearm simulated is positioned at the same pitch angle. This was followed by the steady-state CFD analysis of the hydrodynamic characteristics of a true swimmer's hand model with the thumb in different positions, while the other fingers are kept close together [9]. Drag and lift coefficients were calculated for different angles of attack (the sweepback angle was equal to zero). The combination of drag and lift coefficients (resultant force coefficient) showed that the hand model positioned with the thumb fully abducted presented higher values than the other positions with the thumb partially abducted and adducted at angles of attack of 0° and 45°. However, at an angle of attack of 90°, the position with the thumb adducted presented the highest value of resultant force coefficient. Also, the lift and drag coefficients were steady despite the increase in the flow velocity, and they varied according to changes in pitch angle. Minetti et al. [12] used CFD calculation and in their short communication hypothesized that an intermediate finger spacing in the 3D hand model could increase a higher coefficient of drag providing swimmers with additional thrust. It was indicated that an optimal finger spacing (12°), roughly corresponding to the resting hand posture increases, the drag coefficient (+8.8%), which is functionally equivalent to a greater hand palm area. In the following year, Marinho et al. [10] confirmed these findings using CFD method in their study of the effect of finger spread on the propulsive force production in swimming. The steady-state CFD and 3D scanned models of the hand at the different angles of attack (the sweepback angle equal zero) were used. These results confirmed that the model with a small spread between fingers presented higher values of drag coefficient than did the models with fingers closed and fingers with a large spread. And it was concluded that the optimum finger spread could allow the hand to create more propulsive force during swimming.

Researchers proved that the hand orientation as well as hand shape contributes to the values of hydrodynamics parameters. Rouboa et al. [11] agree that both the propulsion and drag forces will fluctuate during individual phases of stroke cycle along with the respective variation of angle of attack and sweepback angle. Schleihauf (as cited in [13]) summarised that the ideal pitch angle during the underwater motion of the hand will produce an optimal combination of lift and drag forces, which in turn will generate a resultant force that could be predominantly directed in forward direction. Gardano and Dabnichki [7] noticed that, in addition to the pitch angle having the influence on drag force, at the high Reynolds numbers (which swimmers usually undergo in the competitions), two main factors affect the drag force: shape and arm orientation. In previous CFD studies of free style arm strokes, the effect of swimmer's hand orientation and its effect on propulsion force were evaluated while varying exclusively the angle of attack and full consideration of influence of both the angles were seldom considered.

This quasi-steady state (with initial data is taken from real kinematic experimental research) CFD study is based on flow simulation around swimmer's scanned hand models by using FLUENT (ANSYS, PA, USA). It is hypothesize that the changes of hand shape, velocity, and both angles of hand orientation (pitch and sweepback) complement drag force and drag coefficient in underwater freestyle stroke. Also, it is hypothesised that the realistic variation of both the orientation angles contributes to higher values of lift, drag, and resultant coefficients and forces when compared with values from previous work based on CFD method. Therefore, the aim of this study was to evaluate drag force and drag coefficient corresponding to the variation in kinematic parameters (orientation, shape, and velocity) of swimmer's hand during complete underwater front crawl stroke cycle. 

## 2. Methods

In this study, the hydrodynamic components of propulsion force (*F*_*D*_,  *C*_*D*_) using realistic models of human hand were calculated. The FLUENT (ANSYS, PA, USA) software was used to simulate the fluid flow, allowing the analysis of distribution of pressure and flow around the hand model of the swimmer.

### 2.1. Hand's Geometries and Initial Parameters

**Figure 2:**
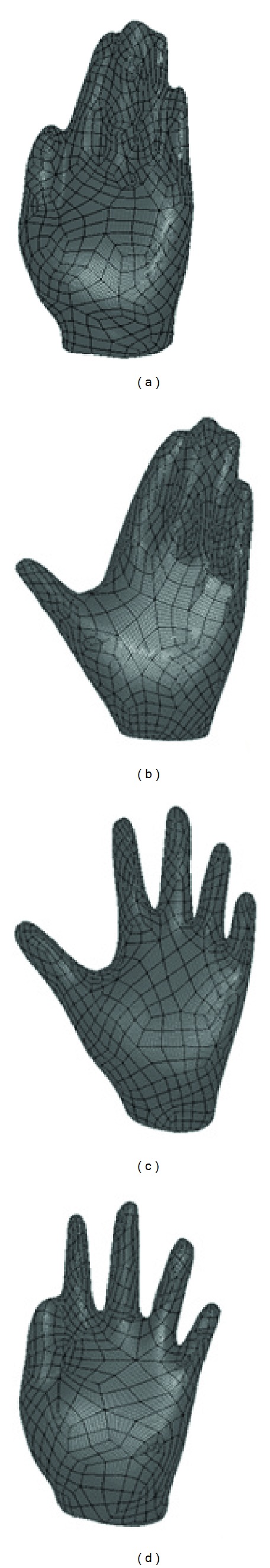
Four different models of swimmer's hand: (a) *H*_adducted_—with thumb adducted; (b) *H*_abducted_—with thumb abducted; (c) *H*_spread_—with spread all fingers; (d) *H*_adducted, spread_—with spreading fingers and thumb adducted.

Artec L 3D scanner (Artec Group Inc., Luxembourg, Lux) was used to scan the left hand of international level swimmer. Working parameters of scanner are video frame rate of up to 15 fps and data acquisition speed up to 500000 points/s. The scanner was directly connected with computer, and all the data was transferred to image-processing program Artec Studio (Artec Group Inc., Luxembourg, Lux). Finally, using this software, cloud point nodal 3D hand models were created from scanned frames: *H*_adducted_—with thumb adducted; *H*_abducted_—with thumb fully abducted; *H*_spread_—with spread fingers; *H*_adducted, spread_—with adducted thumb and spread fingers. To optimize the models and to get good resolution by the selection of relevant parts of model and to transform the point cloud into a complete 3D polygon model, all the data from Artec Studio (Artec Group Inc, Luxembourg, Lux) was transferred to Leios 2 (EGS srl, Bologna, Italy) software. Typical models of hands with 1182, 1204, 1218, and 1142 surfaces corresponding to *H*_adducted_, *H*_abducted_, *H*_spread_ and *H*_adducted, spread_ were created (Figure 2). The Leios2 (EGS srl, Bologna, Italy) software program can export the datasets into IGES (Initial Graphics Exchange Specification) format which allows file import by CFD software FLUENT (ANSYS, PA, USA).

Initial kinematic parameters corresponding to real swimmer's single front crawl underwater stroke cycle were obtained from previous experimental study [13]. The single front crawl stroke cycle is usually divided into four phases: glide—from the entry of the hand into the water to its maximal forward displacement in the longitudinal displacement; pull—from the maximal forward displacement of the hand in the longitudinal displacement (i.e. *Y* direction) corresponding to the stroke time when the hand is located exactly under the shoulder; push—from the end of the pull phase until the exit of the hand out of the water; recovery—from the hand's exit to its reentry into the water [14]. Each model of hand was positioned in nine different positions with the combinations of various pitch (defines angle between hand velocity vector and the plane of the hand) and sweepback angles (which define the inclination of the leading edge of hand) using SolidWorks (Dassault Systémes SolidWorks Corporation, MA, USA) software. Different values of average velocity correspond to different times (Table 1), resulting in three parts of underwater stroke time from glide phase, three from pull and three from push phases. Gourgoulis et al. [13] determined the velocity of the hand as the mean of the resultant velocities of the 2nd and the 5th metacarpophalangeal joints and transformed from the external (O; X, Y, Z) to the local reference system (O; x, y, z) of the swimmer's hand. In CFD calculations, this velocity was used as the resultant average velocity of water flow while the hand model was kept stationary.

### 2.2. Computational Domain

**Figure 3:**
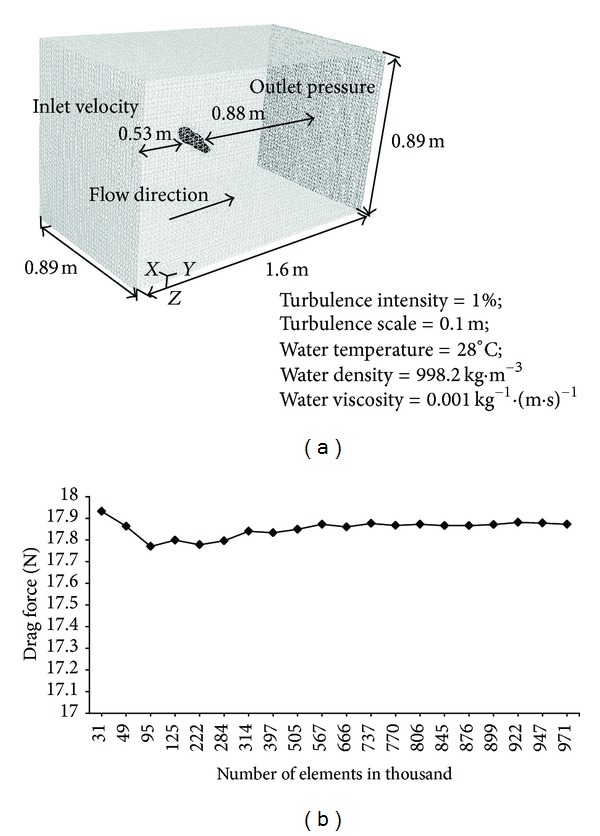
(a) Computational mesh; (b) curve of grid independence test.

All hand positions of four hand models were exported to geometry modelling software DesignModeler (ANSYS, PA, USA) to generate fluid domain and later to Meshing (ANSYS, PA, USA) software to generate mesh (Figure 3(a)). The hand models were placed in the centre of 3D rectangular domain. The flow domain in this study extends from 0.53 m upstream of the hand model up to 0.88 m downstream with full dimensions: length: 1.6 m; width and height are each equal to 0.89 m. To ensure that the model would provide accurate results, the grid was refined in presumed regions of high velocity and pressure gradients.

A series of tests were carried out to estimate the independence of the results in relation to the grid resolution. *H*_adducted_ model was used at hand speed of 1.79 m*·*s^−1^. The pressure force was calculated for each grid (Figure 3(b)). The number of grid elements equal to 844740 was chosen in further calculations to ensure reasonably fast and accurate computations, the independence of grid used, and repeatability of the results.

### 2.3. Computational Fluid Dynamics (CFD)

*Governing Equations*. CFD method was used to simulate drag force and resultant drag coefficient of the hand models. Steady velocity is implemented by keeping hand static with the fluid flowing at constant velocity. CFD method is based on the Navier-Stokes equations which fully govern fluid flow. These equations arise from applying Newton's second law to fluid motion, together with the assumption that the fluid stress is the sum of a diffusing viscous term (proportional to the gradient of velocity), plus a pressure term. The solution of the Navier-Stokes equations is a velocity field or flow field, which is a description of the velocity of the fluid at a given point in space and time. For the incompressible fluids, the continuity equation is only function of velocity and not a function of pressure. Only the momentum equations contain pressure term. A direct method is to discretize the equations of continuity and momentum and solve them simultaneously to obtain results of pressure.

CFD methodology consists of a mathematical model that replaces the Navier-Stokes equations with discretized algebraic expressions that can be solved by algorithms on the finite discretized domain consisting of volumetric mesh with the prediction of fluctuating velocities with the help of turbulent model. The problem of the turbulent modelling was solved using *k*-*ε* model. The system of equations for solving three-dimensional, incompressible fluid flow in steady-state regime is as follows.

Continuity equation

(1) $$\begin{array}{c} \;\frac{\partial }{\partial x_{i}}\left(\bar{U_{i}}\right)=0. \end{array}$$

Navier-Stokes (momentum) equations

(2) ∂∂xj(ρU¯iU¯j) =−∂p¯∂xi+∂∂xj[(μ+μt)(∂∂xj(U¯i)+∂∂xi(U¯j))−23δijρk],

where ${\bar{U}}_{i}\left(t\right)\equiv {\bar{U}}_{i}+{\bar{u}}_{i}$ is the component of instantaneous velocity in *i*-direction (m*·*s^−1^), ${\bar{U}}_{i}$ is the component of time averaged mean velocity in *i*-direction (m*·*s^−1^), *u*_*i*_ is the component of fluctuating velocity in *i*-direction (m*·*s^−1^), *i*, *j* are the coordinate direction vectors, *ρ* is average fluid density (kg*·*m^−3^), *μ* is dynamic viscosity of fluid (kg*·*(m*·*s)^−1^), *μ*_*t*_ is turbulent viscosity of fluid (kg*·*(m*·*s)^−1^), $\bar{p}$ is average pressure (N*·*m^−2^), $k=(1/2)\left({\bar{u}}_{i}{\bar{u}}_{j}\right)$ is the turbulent kinetic energy per unit mass (m^2^ *·*s^−2^), and *δ*_*ij*_ is the Kronecker delta with the condition that, *δ*_*ij*_ = 1 if *i* = *j* and *δ*_*ij*_ = 0 if *i* ≠ *j*.

### 2.4. Boundary Conditions

For the steady state fluid flow simulations, appropriate boundary conditions were considered. On the left side vertical surface of the domain (inlet velocity, Figure 3(a)), the horizontal component of the initial velocity was applied for all hand positions, respectively, (Table 1) and the vertical component of the velocity was assumed to be equal to zero. The pressure was set equal to zero Pascal on the right side of vertical surface (outlet pressure, Figure 3(a)). The remaining side surfaces and bottom of the domain were considered as symmetry. Incompressible flow was assumed with turbulence intensity of 1.0% and turbulence scale of 0.10 m. The water temperature was 28°C with a density of 998.2 kg*·*m^−3^ and viscosity of 0.001 kg^−1^ *·*(m*·*s)^−1^ with consideration of the gravity of 9.81 m*·*s^−2^. 

### 2.5. Numerical Scheme

More accurate solution was considered with the choice of second-order numerical computational schemes. The simulations are based on finite volume method of discretization. In generic terms, the convergence of the calculation is checked by the value of the residuals of the various flow parameters. The convergence criteria in FLUENT (ANSYS, PA, USA) were set at 10^−6^. This criterion is assumed sufficient to ensure the convergence of the solution for the present study. The appropriate number of tetrahedral grids cells in the simulation model was arrived, which was an outcome of grid independence test carried out at the beginning of actual simulations. It was found that the difference in solutions for the drag coefficients for subsequent refinement in tetrahedral grid was less than 1%. In order to limit numerical dissipation, particularly when the geometry is complex consisting of an unstructured grid, as seen in Figure 3(a), the choice of second-order upwind discretization scheme for the convection terms in the solution equations and Pressure-Implicit with Splitting of Operators (PISO) pressure-velocity coupling scheme for the double precision pressure-based solver was chosen. The PISO pressure-velocity coupling scheme, part of the SIMPLE family of algorithms, is based on the higher degree of the approximate relation between the corrections for pressure and velocity.

## 3. Results 

The calculated values of drag forces, drag coefficients, and areas of hands of all the studied phases at three different velocities (differences from initial velocity ± 0.5 m*·*s^−1^) are presented in Tables 2 and 3, along with the corresponding data of all hand models used. The largest mean of drag force (40.85 N, *s* = 18.02 N) by *H*_adducted_ was observed during pull phase, which also presented the largest projected hand area, whereas hand models *H*_abducted_, *H*_spread_, *H*_adducted,  spread_ (appropriately: 43.95 N, *s* = 4.56 N; 51.08 N, *s* = 2.04 N; 41.51 N, *s* = 2.80 N) presented largest mean of drag force during the push phase, when the initial mean velocity was greater than before. The lowest drag forces of all hand models were during the glide phase when initial flow velocity was decreased 0.5 m*·*s^−1^ and projected hand area was the smallest. The maximum values of *C*_*D*_ were calculated during the pull phase of all hand models (mean values: 1.96, *s* = 0.36, 1.84, *s* = 0.28, 1.65, *s* = 0.07, and 1.75, *s* = 0.19, accordingly *H*_adducted_, *H*_abducted_, *H*_spread_, and *H*_adducted, spread_), when flow velocity was the lowest. Minimum mean values of drag coefficients were reached during the glide phase, when initial flow velocity was increased 0.5 m*·*s^−1^ and projected hand area was the smallest.

**Figure 4:**
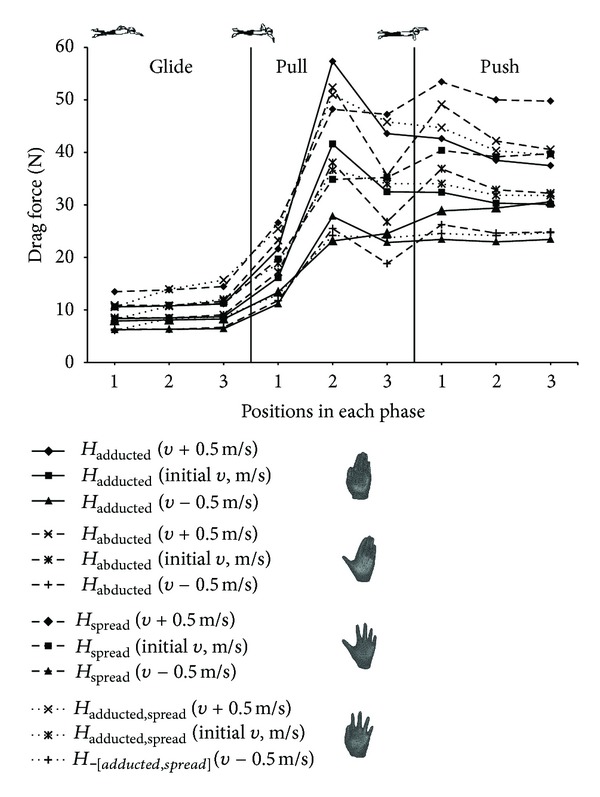
Drag force versus different hand position acting on separated swimmer's hand models corresponding to three different phases with respective orientations and increment of velocity.

**Figure 5:**
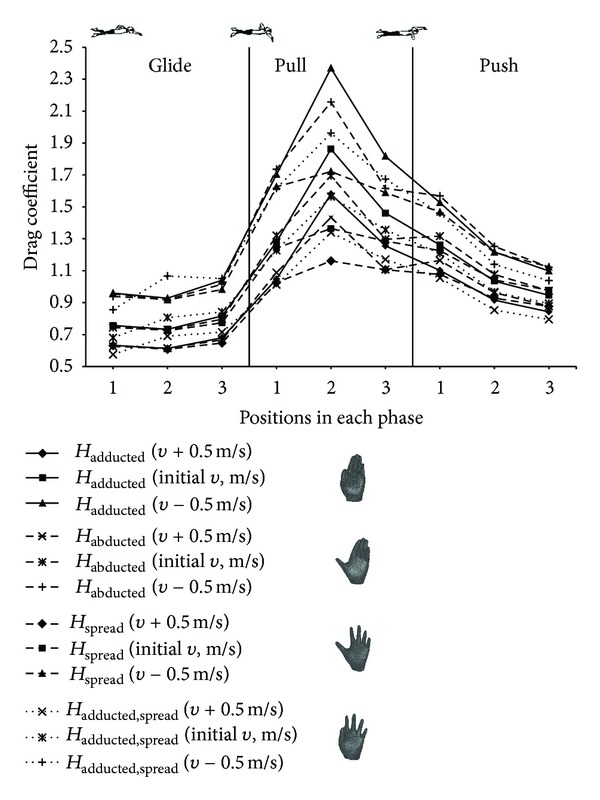
Drag coefficient versus different hand position acting on separated swimmer's hand models corresponding to three different phases with respective orientations and increment of velocity.

A variation of drag force and drag coefficient is observed due to different hand orientation, which depends on both hand angles. *F*_*D*_ slightly varied during the glide phase (Figure 4). All drag forces rose in the beginning of pull phase and reached maximum values in the middle of this phase when projected hand area was the biggest (except *H*_spread_ max *F*_*D*_ during the first point of push phase). The values of the drag forces fell after peaks due to decrease in projected hand area. *F*_*D*_ varied more when initial velocity was increased, and there was less variation when initial velocity was decreased. Drag coefficient slightly varied in glide phase (Figure 5). The values of *C*_*D*_ rapidly increased from the 3rd position of the glide phase to the 2nd position of the pull phase due to significant changes in hand angles which in turn affected projected hand area. Drag coefficients decreased in push phase of all hand models.

There is a marked improvement in drag force and drag coefficient changes due to different shape of hand (Figures 4 and 5). *F*_*D*_ was not significant during the glide phase; however, the biggest means were observed in *H*_spread_ and *H*_adducted, spread_. *H*_adducted_ and *H*_abducted_ reached maximum peaks in the pull phase though the values of drag force were not the biggest during push phase. All drag forces of *H*_adducted_, *H*_abducted_, and *H*_adducted, spread_ slightly decreased in push phase initially and increased initial velocities and were almost stable during decreased initial velocity. Meanwhile, *F*_*D*_ of *H*_spread_ was negligibly increasing in push phase during decreased velocity and slightly varied during the initial and increased initial velocities. Drag coefficient varied insignificantly during the glide phase. Significant variation was observed in push phase of all hand models. *H*_adducted_ reached the biggest values of *C*_*D*_ in the pull phase; however, values of *C*_*D*_ were the biggest of *H*_abducted_ in push phase for every variation in velocity.

**Figure 6:**
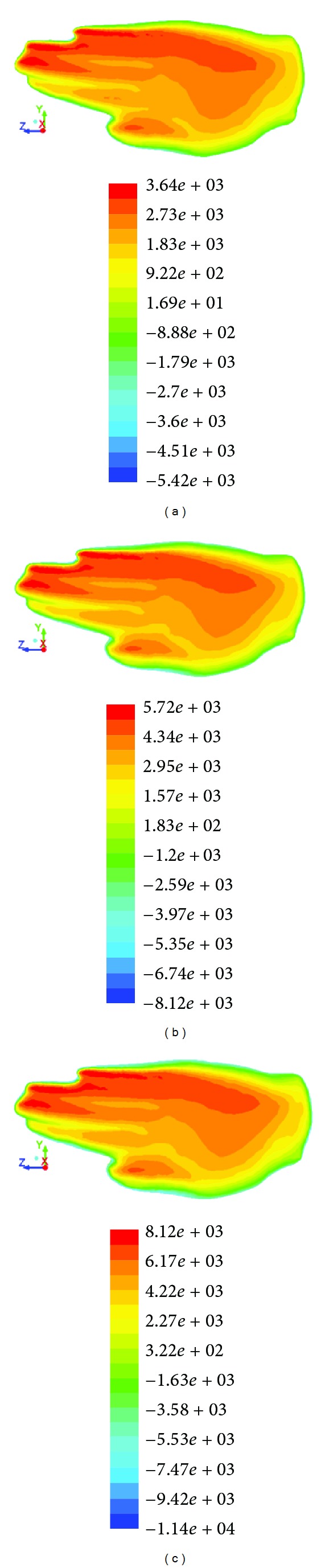
Static pressure (Pascal) acting on the same swimmer's hand model—*H*_adducted_, at the same underwater phase with regard to different water flow velocity: (a) 1.32 m*·*s^−1^, (b) 1.82 m*·*s^−1^, and (c) 2.32 m*·*s^−1^.

**Figure 7:**
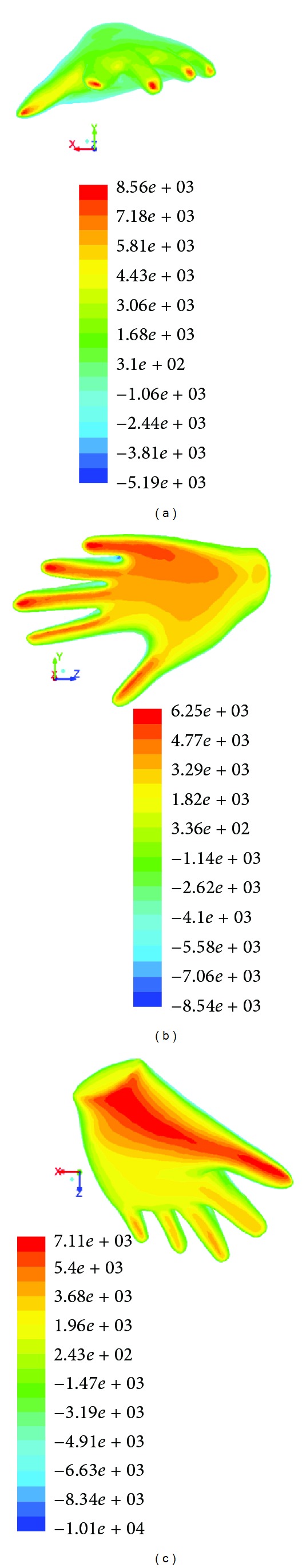
Static pressure (Pascal) acting on the same swimmer's hand model—*H*_spread_, corresponding to different orientations at three underwater phases: (a) glide, (b) pull, and (c) push.

**Figure 8:**
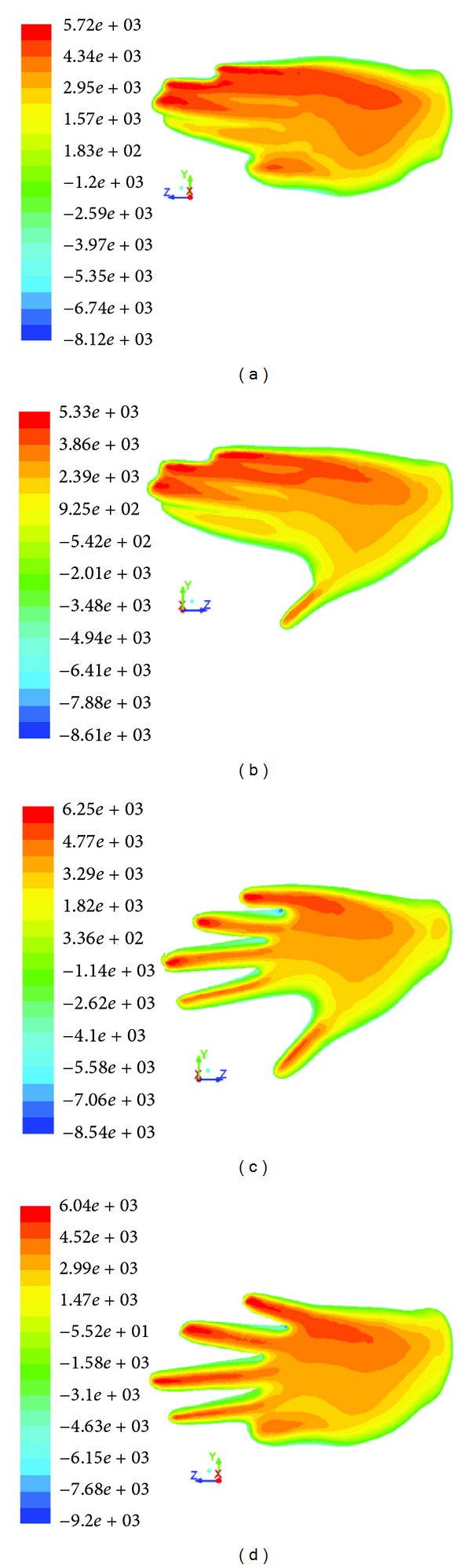
Static pressure (Pascal) acting at the same water flow velocity (1.82 m*·*s^−1^) of 2nd pull position on separated swimmer's hand models: (a) *H*_adducted_—with thumb adducted; (b) *H*_abducted_—with thumb abducted; (c) *H*_spread_—with spread all fingers; (d) *H*_adducted, spread_—with spread fingers and thumb adducted.

Pressure forces contribution to hand varied according to water flow velocity, hand orientation, and shape. Pressure visualisation considering different water flow velocity (1.82 ± 0.5 m*·*s^−1^) was proposed on *H*_adducted_ during 2nd position of pull phase (Figure 6). Pressure contour fields depending on different hand orientation of *H*_spread_ during separated underwater freestyle phases were shown (Figure 7). The shape of hand caused different pressure force for the same water flow velocity (1.82 m*·*s^−1^) during 2nd position of pull phase (Figure 8).

**Figure 9:**
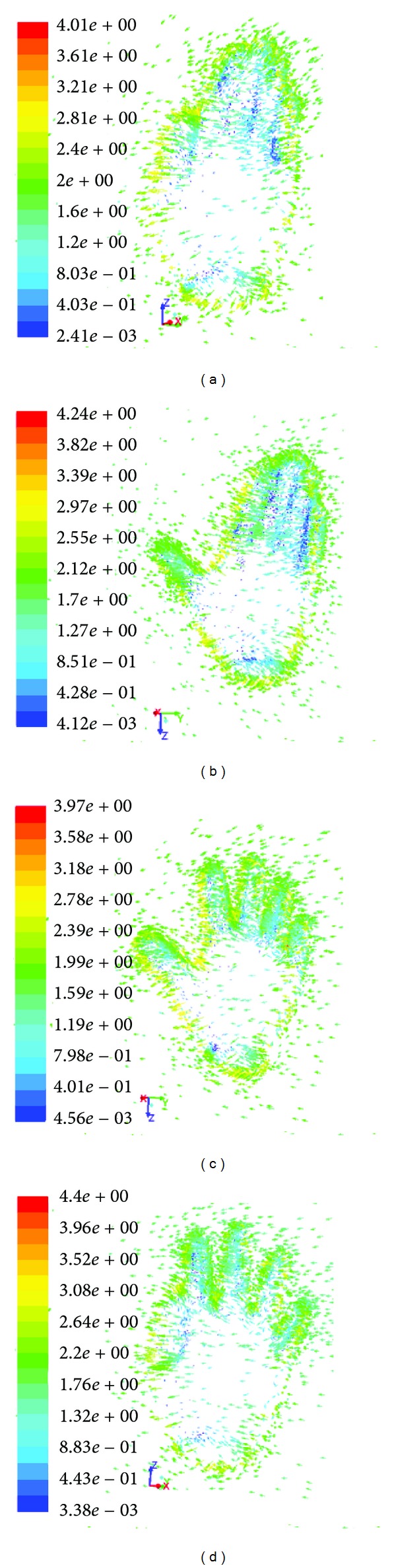
Velocity vectors on the different models of the hand: (a) *H*_adducted_, (b) *H*_abducted_, (c) *H*_spread_, and (d) *H*_adducted, spread_.

The vectors of flow velocity distribution on the palm of the hand are presented (Figure 9). The velocity of flow decreased when in contact with hand surface. Flow dispersed to the corners of the palm with increasing in velocity. The biggest values of mean fluid velocity were observed near the edges of the palm of the hand and between the edges of fingers in case of hand model with spread fingers. 

## 4. Discussion

The purpose of this study was to evaluate drag force and drag coefficient correspondence to variation in kinematic parameters (orientation, shape, and velocity) of swimmer's hand during complete underwater front crawl stroke cycle. Four scanned hand models with fingers in different positions were generated. The orientation of hand was set into nine different positions with accordance to average velocity. In the present study, the analysis of a particular case of front crawl underwater movement (Table 1) and initial kinematic data were taken from experimental kinematic research work [13]. Calculations were performed through FLUENT (ANSYS, PA, USA) software, which is based on computational fluid dynamics method applied to accurately solve fluid flow problems through numerical simulation. This method has been proven to generate accurate results with repeatability of results with identical values, when performed with similar initial conditions and settings [17]. This saves time, and also the results can be accessed in detail and analysed anytime, unlike repetitive experimental tests. The outcome of this study is that the modification of three variables: hand orientation, shape, and the average water flow velocity strongly affects drag coefficient and drag force of hand and with this in some measures affects the hand propulsion.

There is clear indication of progress in the understanding of the contribution of hand shape, orientation, and velocity on drag coefficient (*C*_*D*_) and drag force (*F*_*D*_) in underwater hand stroke. Obvious drag force and drag coefficient dependence on velocity are shown in Figures 4 and 5. The values of *F*_*D*_ are seen to increase and decrease with respect to the initial velocity, which was accordingly increased and decreased in 0.5 m*·*s^−1^intervals. A similar tendency of increase of *F*_*D*_ with velocity was observed in a previous study performed under steady-state flow conditions of the 2D and 3D swimmer's hand model [11, 18]. Rouboa et al. [11] simulated a hand/forearm model with thumb adducted in three different orientations where water flow velocity was increased from 0.5 m*·*s^−1^ to 4 m*·*s^−1^. Sato and Hino [18] used hand model with spread fingers in a fixed direction with water flow, and velocity was set from 0.5 m*·*s^−1^ to 2 m*·*s^−1^. However, the variation of current drag coefficient (Figure 5) and previous findings disagree. It was indicated that the drag coefficient did not vary, while there was rise in water flow velocity [9, 11, 18]. Berger et al. [6] towed model of human hand in a towing tank at the similar orientation and evaluated hydrodynamic parameters. It was shown that the *C*_*D*_ slightly decreased within the velocity range from 0.7 to 3.0 m*·*s^−1^, and at the velocity lower than 0.7 m*·*s^−1^ *C*_*D*_ strongly depends on velocity. Current findings indicated that the rise in velocity generated lower values of drag coefficients for all hand models during all phases. According to current findings, it can be concluded that the velocity affects values of drag force and drag coefficient during all front crawl underwater phases.

To our knowledge, there is no known work based on CFD method which compares different phases of underwater front crawl hand motion with appropriate changes of hand orientation. However, Gourgoulis et al. [19] calculated drag force of human hand by formulas while input data was taken from kinematic experiments. The drag force dependence on hand orientation appears with the variation of *F*_*D*_ in both current (Figure 4) and previous research. There is clear indication that, between different phases of underwater hand movement, when swimmer changes his hand orientation (i.e., pitch and sweepback angle) (Table 1), the changes directly affect the projected frontal area and hereupon the drag force. The variation of *F*_*D*_ calculated from experimental data was similar with current computational hand model *H*_abducted_ with similar initial flow velocity. Therefore, we could imply that the swimmer maintained his hand with thumb abducted during underwater hand stroke experimental tests.

The calculation of drag coefficient during different underwater phases of hand model allowed evaluating the influence of hand orientation on *C*_*D*_. This study showed that small changes in pitch and sweepback angles during glide phase caused negligible variation of *C*_*D*_ for all hand models (Figure 5). The sharp variation of hand angles during the pull phase caused major changes in values of *C*_*D*_, and the dwindling of angles to smaller values shaped the reduction of drag coefficient during push phase. The consideration of the angle of attack and sweepback angle influenced contributions to drag coefficient during complete underwater cycle by different hand models.

The importance of hand shape variation and its contributory influence to drag force are presented (Figure 4). Different individual curve lines in the pictorial graph represent separate models of the swimmer's hand. The highest peak value of drag force was obtained from the hand model with thumb adducted and the smallest peak from the model with all fingers spread in the middle of the pull phase regardless of variation in flow velocity. Although the hand area, that directly affects the drag force, was larger in the model with fingers spread than in that with the thumb adducted, the pitch angle was the largest in this position. However, in the end of pull phase and throughout the push phase, when the hand altered its leading edge, the hand with spread fingers generated the biggest projected area of total plane area of hand and values of *F*_*D*_, whereas the hand with the thumb adducted—the least. It means that the shape of the hand had different contributions on the drag force during separate underwater phases of front crawl stroke cycle. Likewise, it was summarised that higher drag force values could be reasonable by a hand model with optimal spacing between fingers [10, 12]. According to the current results, it is clear that, in order to increase the drag force contribution throughout the underwater stroke cycle, the shape of the hand should be altered in separate individual phases.

The drag coefficient values (Figure 5) corresponding to different hand models were evaluated in recent CFD studies under steady state with spread fingers [10, 12, 18], with thumb in different positions [9], while current study used different values of velocity, pitch angle, and also different shapes of hand models. In previous studies, the drag coefficient was almost constant despite the changes in velocity. We observed that the drag coefficient was not constant and it varied throughout underwater front crawl cycle regardless of differences in hand model.

**Figure 10:**
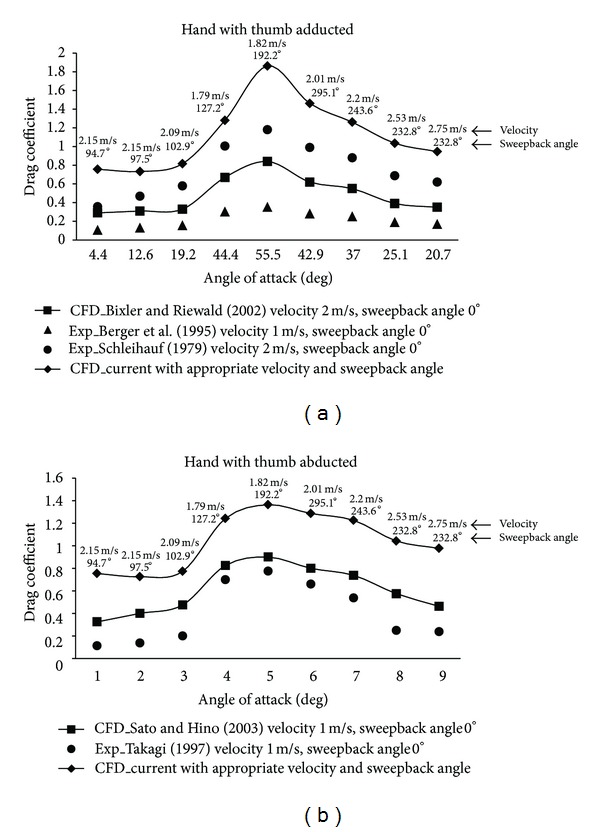
Comparison of drag coefficient versus angle of attack acting on swimmer's hand: (a) current CFD results (with appropriate sweepback angle and flow velocity in each phase), Bixler and Riewald [5] CFD (velocity 2 m·s^−1^, sweepback angle 0°), Berger et al. [6] experimental results (velocity 1 m·s^−1^, sweepback angle 0°) and Schleihauf [15] experimental results (velocity 2 m·s^−1^, sweepback angle 0°); (b) current CFD study (with appropriate sweepback angle and flow velocity in each phase), Sato and Hino [12] CFD, and Takagi [16] (cited in [12]) experimental results where flow velocity is 1 m·s^−1^ and sweepback angle is 0°.

In the current study, we compared drag coefficients from the hand model with thumb adducted with previous studies obtained experimentally and from CFD studies, which were achieved by varying only the angle of attack in appropriate range while sweepback angle was equal to zero [5, 6]. The comparative study of current and previous *C*_*D*_ values versus angle of attack (Figure 10(a)) showed the similar tendency of variation in *C*_*D*_ (the hydrodynamic characteristics between all research work were similar). However, the means of current results were higher when compared with previous ones. These perceived differences could be due to differences in the usage of distinct input conditions, and, according to Berger et al. [6], material, hand size, and shape of the model might also account for the observed differences in *C*_*D*_. Moreover, the distinct tendency of variation can also be caused by different hand orientation, which considered variation of both pitch and sweepback angles at the same time corresponding to that observed under real swimming conditions during underwater hand stroke path.

The drag coefficients of hand model with fingers spread obtained in this study were compared (Figure 10(b)) with those in Takagi's (as cited in [18]) study, obtained from experimental research, and with those in Sato and Hino's work [18] calculated through CFD method. The kinematic data from experimental work [13] and scanned human hand model were used for this study to evaluate drag force and drag coefficient; therefore, *C*_*D*_ values and its variation present practical and further pragmatic results. Obvious differences are observed in values of the drag coefficients between present and previous CFD and experimental studies; whereas the tendency of variation was similar. Higher *C*_*D*_ values in current work could be influenced by the choice of fluid flow model applied in the study: turbulent fluid flow was considered in this study, and laminar fluid flow was considered by Sato and Hino [18]. The values of *C*_*D*_ were significantly higher in this study as the *C*_*D*_ was dependent on not only the pitch angle, as considered in previous studies, but on both angles of hand orientation (pitch and sweepback). Furthermore, the consideration of experimental kinematic data of swimmer's hand motion (initial velocity, pitch, and sweepback angles) and realistic fluid flow model can affect realistic estimation of drag coefficient.

It was shown that the pressure force contribution on swimmer's hand acted differently vis-à-vis water flow velocity, hand orientation, and shape. The results of this study confirmed that the velocity of water flow directly affected pressure force on swimmer's hand (Figure 6). The contribution of pressure force to hand increased when velocity was increased. There is strong evidence that the pressure force affected different parts of hand due to altered hand sweepback angle and bigger part of hand frontal cross area came up with maximum pressure force, when pitch angle was increased (Figure 7). This maximal pressure force shifted towards hand's leading edge; this is when the swimmer altered orientation of the hand, that is, the angle of attack and sweepback angle. Different hand shapes arrived at different pressure force distributions (Figure 8). In this case, the biggest maximal value of pressure (6250 Pa) acted on hand with spread fingers (Figure 8(c)) and the least (5720 Pa)—on hand with thumb adducted (Figure 8(a)). The present study corroborates with Marinho et al. [10] and Minetti et al. [12], with reference to the maximum values of the pressure force, suggesting that the hand with spread fingers goes through higher pressure force and in turn influences higher drag force than other forms of hand model.

## 5. Conclusions

This study, based on the CFD method, indicated that drag force and drag coefficient were significantly affected by velocity, hand frontal cross-section area, and both angles of hand orientation (pitch and sweepback) during complete underwater front crawl single cycle. Higher values of hydrodynamic parameters in this study are attributed to the consideration of real swimming conditions during simulations with the consideration of combination of both hand orientation angles observed during individual phases. To increase drag force contribution throughout the underwater stroke, the shape of the hand should be altered in separate phases.

## Figures and Tables

**Table 1:** Initial experimental kinematic data (Gourgoulis et al. [13]) applied in steady state CFD calculations.

Phases	Time (%)	Velocity (m·s^−1^)	Angles of attack (deg)	Sweepback angle (deg)
	10	2.15	4.44	94.75
Glide	20	2.15	12.57	97.45
	30	2.09	19.23	102.87

	55	1.79	44.37	127.23
Pull	65	1.82	55.46	192.20
	75	2.01	42.89	295.06

	85	2.20	36.98	243.63
Push	90	2.53	25.14	232.80
	95	2.75	20.71	232.80

**Table 2:** Values of the areas, drag forces, and drag coefficients at different increase of velocity in separated underwater phases varied according to the model of hand: *H*_adducted_ and *H*_abducted_.

Phases	*H*_adducted_ area (×10^−3^ m^2^)	Initial velocity, m·s^−1^		Initial velocity −0.5 m·s^−1^		Initial velocity +0.5 m·s^−1^		*H*_abducted_ area (×10^−3^ m^2^)	Initial velocity, m·s^−1^		Initial velocity −0.5 m·s^−1^		Initial velocity +0.5 m·s^−1^	
		*F*_*D*_(*N*)	*C*_*D*_	*F*_*D*_(*N*)	*C*_*D*_	*F*_*D*_(*N*)	*C*_*D*_		*F*_*D*_(*N*)	*C*_*D*_	*F*_*D*_(*N*)	*C*_*D*_	*F*_*D*_(*N*)	*C*_*D*_
Glide 1	4.77	8.33	0.757	6.22	0.959	10.57	0.633	4.97	8.53	0.744	6.34	0.939	10.89	0.626
Glide 2	5.01	8.48	0.733	6.32	0.928	10.78	0.613	5.03	8.46	0.729	6.28	0.920	10.83	0.614
Glide 3	4.90	8.71	0.816	6.43	1.040	11.14	0.680	5.22	9.07	0.796	6.69	1.015	11.66	0.667

Mean	4.89	8.51	0.77	6.32	0.98	10.83	0.64	5.07	8.69	0.76	6.44	0.96	11.13	0.64

*s*	0.12	0.19	0.04	0.10	0.06	0.29	0.03	0.13	0.33	0.04	0.22	0.05	0.46	0.03

Pull 1	7.89	16.15	1.280	11.16	1.703	21.62	1.047	8.16	17.23	1.320	11.77	1.736	23.24	1.088
Pull 2	13.51	41.59	1.862	27.83	2.369	57.36	1.580	13.60	38.06	1.694	25.50	2.156	52.35	1.433
Pull 3	11.04	32.50	1.461	22.83	1.817	43.58	1.256	10.24	26.79	1.297	18.82	1.616	35.64	1.107

Mean	10.81	30.08	1.53	20.60	1.96	40.85	1.29	10.67	27.36	1.44	18.70	1.84	37.08	1.21

*s*	2.82	12.89	0.30	8.56	0.36	18.02	0.27	2.74	10.43	0.22	6.86	0.28	14.61	0.19

Push 1	10.63	32.40	1.261	23.42	1.527	42.63	1.102	11.60	36.87	1.316	26.24	1.569	49.13	1.164
Push 2	9.17	30.32	1.035	22.97	1.218	38.48	0.915	9.56	32.89	1.077	24.63	1.252	42.18	0.963
Push 3	8.44	30.12	0.946	23.41	1.098	37.48	0.842	8.77	32.23	0.974	24.85	1.122	40.55	0.877

Mean	9.42	30.95	1.08	23.27	1.28	39.53	0.95	9.97	34.00	1.12	25.24	1.31	43.95	1.00

*s*	1.12	1.26	0.16	0.26	0.22	2.73	0.13	1.46	2.51	0.18	0.87	0.23	4.56	0.15

**Table 3:** Values of the areas, drag forces, and drag coefficients at different increase of velocity in separated underwater phases varied according to the model of hand: *H*_spread_ and *H*_adducted,spread_.

Phases	*H*_spread_ area (×10^−3^ m^2^)	Initial velocity, m·s^−1^		Initial velocity −0.5 m·s^−1^		Initial velocity +0.5 m·s^−1^		*H*_adducted,spread_ area (×10^−3^ m^2^)	Initial velocity, m·s^−1^		Initial velocity −0.5 m·s^−1^		Initial velocity +0.5 m·s^−1^	
		*F*_*D*_(*N*)	*C*_*D*_	*F*_*D*_(*N*)	*C*_*D*_	*F*_*D*_(*N*)	*C*_*D*_		*F*_*D*_(*N*)	*C*_*D*_	*F*_*D*_(*N*)	*C*_*D*_	*F*_*D*_(*N*)	*C*_*D*_
Glide 1	6.09	10.59	0.754	7.90	0.955	13.46	0.631	5.24	8.21	0.680	6.09	0.855	10.55	0.575
Glide 2	6.50	10.86	0.725	8.09	0.916	13.82	0.607	5.76	10.72	0.806	8.35	1.067	13.94	0.690
Glide 3	6.67	11.24	0.773	8.27	0.982	14.45	0.647	6.55	12.02	0.842	8.69	1.051	15.68	0.715

Mean	6.42	10.90	0.75	8.09	0.95	13.91	0.63	5.85	10.32	0.78	7.71	0.99	13.39	0.66

*s*	0.30	0.33	0.02	0.18	0.03	0.50	0.02	0.66	1.94	0.09	1.41	0.12	2.61	0.07

Pull 1	9.91	19.68	1.242	13.38	1.626	26.60	1.026	9.61	18.86	1.227	12.90	1.616	25.46	1.012
Pull 2	15.46	34.86	1.364	23.14	1.721	48.22	1.161	14.17	36.65	1.564	24.18	1.962	50.99	1.339
Pull 3	13.58	35.22	1.286	24.56	1.589	47.23	1.106	12.45	34.03	1.356	23.72	1.674	45.80	1.170

Mean	12.98	29.92	1.30	20.36	1.65	40.69	1.10	12.08	29.85	1.38	20.26	1.75	40.75	1.17

*s*	2.82	8.87	0.06	6.09	0.07	12.21	0.07	2.31	9.60	0.17	6.39	0.19	13.49	0.16

Push 1	13.63	40.38	1.226	28.86	1.467	53.43	1.077	11.66	34.03	1.208	24.56	1.461	44.71	1.054
Push 2	11.77	39.17	1.042	29.40	1.215	50.02	0.928	10.31	31.85	0.967	24.16	1.139	40.30	0.853
Push 3	10.78	39.74	0.977	30.59	1.124	49.78	0.876	9.43	31.81	0.894	24.72	1.037	39.52	0.795

Mean	12.06	39.76	1.08	29.62	1.27	51.08	0.96	10.47	32.56	1.02	24.48	1.21	41.51	0.90

*s*	1.45	0.61	0.13	0.89	0.18	2.04	0.10	1.12	1.27	0.16	0.29	0.22	2.80	0.14
